# At-home computerized executive-function training to improve cognition and mobility in normal-hearing adults and older hearing aid users: a multi-centre, single-blinded randomized controlled trial

**DOI:** 10.1186/s12883-023-03405-1

**Published:** 2023-10-20

**Authors:** Rachel Downey, Nathan Gagné, Niroshica Mohanathas, Jennifer L. Campos, Kathleen M. Pichora-Fuller, Louis Bherer, Maxime Lussier, Natalie A. Phillips, Walter Wittich, Nancy St-Onge, Jean-Pierre Gagné, Karen Li

**Affiliations:** 1 Department of Psychology, Concordia University, Montréal, Québec Canada; 2 PERFORM Centre, Concordia University, Montréal, Québec Canada; 3 Department of Psychology, University of Toronto, Toronto, ON Canada; 4 KITE-Toronto Rehabilitation Institute, University Health Network, Toronto, ON Canada; 5 Département de Médecine, Université de Montréal, Montréal, Québec Canada; 6 Centre de Recherche de L’Institut de Cardiologie de Montréal, Montréal, Québec Canada; 7 Centre de Recherche de L’Institut Universitaire de Gériatrie de Montréal, Montréal, Québec Canada; 8 École d’optométrie, Université de Montréal, Montréal, Québec Canada; 9 Department of Health, Kinesiology and Applied Physiology, Concordia University, Montreal, QC Canada; 10 École d’orthophonie Et d’audiologie, Université de Montréal, Montréal, Québec Canada

**Keywords:** Hearing loss, Hearing aids, Aging, Falls, Dual-task, Executive function, Cognitive training, Prevention, Neuroimaging, Virtual reality

## Abstract

**Background:**

Hearing loss predicts cognitive decline and falls risk. It has been argued that degraded hearing makes listening effortful, causing competition for higher-level cognitive resources needed for secondary cognitive or motor tasks. Therefore, executive function training has the potential to improve cognitive performance, in turn improving mobility, especially when older adults with hearing loss are engaged in effortful listening. Moreover, research using mobile neuroimaging and ecologically valid measures of cognition and mobility in this population is limited. The objective of this research is to examine the effect of at-home cognitive training on dual-task performance using laboratory and simulated real-world conditions in normal-hearing adults and older hearing aid users. We hypothesize that executive function training will lead to greater improvements in cognitive-motor dual-task performance compared to a wait-list control group. We also hypothesize that executive function training will lead to the largest dual-task improvements in older hearing aid users, followed by normal-hearing older adults, and then middle-aged adults.

**Methods:**

A multi-site (Concordia University and KITE-Toronto Rehabilitation Institute, University Health Network) single-blinded randomized controlled trial will be conducted whereby participants are randomized to either 12 weeks of at-home computerized executive function training or a wait-list control. Participants will consist of normal-hearing middle-aged adults (45–60 years old) and older adults (65–80 years old), as well as older hearing aid users (65–80 years old, ≥ 6 months hearing aid experience). Separate samples will undergo the same training protocol and the same pre- and post-evaluations of cognition, hearing, and mobility across sites. The primary dual-task outcome measures will involve either static balance (KITE site) or treadmill walking (Concordia site) with a secondary auditory-cognitive task. Dual-task performance will be assessed in an immersive virtual reality environment in KITE’s StreetLab and brain activity will be measured using functional near infrared spectroscopy at Concordia’s PERFORM Centre.

**Discussion:**

This research will establish the efficacy of an at-home cognitive training program on complex auditory and motor functioning under laboratory and simulated real-world conditions. This will contribute to rehabilitation strategies in order to mitigate or prevent physical and cognitive decline in older adults with hearing loss.

**Trial registration:**

Identifier: NCT05418998. https://clinicaltrials.gov/ct2/show/NCT05418998

## Background

There is a high prevalence of hearing impairment in older adults, with up to two-thirds of adults aged 70 years and older having bilateral peripheral hearing loss [1, 2]. Epidemiological studies have demonstrated an association between hearing loss and cognitive decline [3], incident dementia [4–7], frailty and increased risk of falling [8, 9], poor balance [10], and reduced gait speed [11]. Several mechanistic theories have been proposed to explain the association between hearing loss and cognitive decline (e.g., sensory deprivation, common cause, effortful listening) [12, 13]. Effortful listening theories argue that when an auditory signal is impoverished due to hearing loss, increased attentional resources must be allocated to hearing, drawing on the same resources needed for other ongoing tasks requiring higher-level cognitive processing [12, 14, 15]. As higher level cognition is involved in the regulation of posture and gait in old age [16], competition for cognitive resources due to hearing loss can therefore lead to reduced performance on mobility tasks requiring attention.

### Empirical evidence supporting effortful listening

Substantial evidence exists in support of effortful listening theories, including studies that compare older adults with normal hearing versus older adults with hearing loss, as well as those that manipulate the level of cognitive demand or listening effort. For example, in studies where the acoustic challenge is increased (e.g., degraded speech, speech-in-noise tasks), normal-hearing individuals exhibit greater difficulty processing linguistically complex sentences, poorer speech comprehension, and reduced memory for auditory information [17]. Additionally, studies utilizing a dual-task paradigm where a cognitive load is added to a mobility task have shown that older adults with hearing loss tend to prioritize posture or walking over cognitive performance (i.e., they have greater dual-costs in the cognitive domain) [18, 19]. In concordance with effortful listening theories, this may be due to the increased attentional resources needed by older adults with hearing loss to process the auditory signal, thereby causing competition for higher level cognitive processes required during dual-tasking and promoting postural prioritization to maintain safe mobility. Finally, neuroimaging studies also lend support for this theory, with increased cortical activation found in regions implicated in cognitive control (e.g., prefrontal cortex) during effortful listening in both normal-hearing adults [20, 21] and older adults with hearing loss [22–24]. Taken together, this evidence supports effortful listening theories insofar as higher-level cognitive resources are needed to compensate for impoverished auditory inputs.

### Ecologically valid measurements of hearing, cognition, and mobility

While these findings elucidate a possible mechanism underlying the association between hearing loss and cognitive and physical decline, some researchers have argued that the typical laboratory-based approaches used to measure hearing, cognition, and mobility lack ecological validity [25, 26]. One method to address this issue is the use of immersive, realistic, multisensory Virtual Reality (VR) environments, which may more accurately estimate a person’s cognitive, listening, or physical abilities during everyday behaviours e.g., [19, 27–29]. Moreover, while behavioural measures of hearing ability, like pure-tone audiometry, are useful for identifying the degree of hearing loss, self-reported hearing measures may be more ecologically valid. Indeed, self-reported hearing measures often consider functional difficulties, such as fatigue due to listening effort, across a variety of settings, which may more accurately reflect everyday listening difficulties [30]. Utilizing such techniques is therefore critical to enhance the ecological validity of hearing, cognitive, and mobility measures.

### Interventions for hearing loss

Hearing loss can begin in mid-life and has been reported to be the potentially largest modifiable risk factor for dementia [5, 6]. Hearing aids are a readily available rehabilitation strategy used to amplify the auditory signal at differing frequencies in order to restore the audibility of sound, with demonstrated benefits in terms of improved communication [31–33]. Evidence suggests that hearing aids may improve executive functioning [34] and speech-in-noise perception [35]. However, more randomized controlled trials are needed to corroborate the possible benefits of hearing aids on cognition. Similarly, the current evidence for the effect of hearing aids on mobility is mixed, with a need for more well-designed randomized controlled trials [36]. In addition to hearing aids, auditory training may be used as a rehabilitation strategy to improve speech perception through bottom-up (i.e., sensory refinement of sounds) or top-down (i.e., cognitive control) processes. A recent meta-analysis examining the pooled effect of auditory training on cognition in adults with hearing loss showed that auditory training led to transfer of learning to untrained cognitive tasks, particularly working memory and overall cognition [37]. However, none of the reviewed studies considered motor outcomes. As such, investigations into other interventions to prevent or mitigate cognitive and physical decline in older adults with hearing loss are warranted.

Improving higher level cognition through executive function training may be particularly beneficial as it directly targets the theoretical mechanisms of effortful listening. Accumulating evidence suggests that in normal-hearing older adults, executive function training can improve cognitive, motor, and dual-task performance [38–45]. While more limited, there is also some evidence to suggest that combined exercise and cognitive training may benefit cognitive and motor functioning in older adults with hearing loss [46, 47]. Li et al. [16] propose that cognitive interventions may increase compensatory scaffolding in response to declining motor and sensory systems with age via up-regulation of cortical activity in regions responsible for higher level cognitive functioning. However, the effect of executive function training on brain activity during dual-tasking in older adults with hearing loss has yet to be examined. Additionally, while some indicators of improved cognitive/mobility-related performance have been observed following lab-based training protocols, the broad application and generalizability of these training protocols is constrained. Instead, at-home cognitive training interventions would allow for more broadly accessible and scalable solutions for cognitive training. Indeed, the COVID-19 pandemic accelerated the development and use of tele-health applications increasing the feasibility of these approaches.

### Research objectives and hypotheses

Given the association between hearing loss and cognitive and physical impairment, there is great value in identifying treatment strategies targeted at delaying or mitigating further decline. The primary objective of our research is to establish the efficacy of an at-home computerized executive function training program on complex auditory and motor functioning measured under both laboratory and simulated real-world conditions. Behavioural and neuroimaging data will be collected in middle-aged and older adults with normal hearing sensitivity thresholds, as well as older hearing aid users.

It is hypothesized that executive function training will lead to greater improvements in dual-task performance compared to no training (i.e., wait-list control group). Furthermore, it is hypothesized that the greatest improvements will be seen in older hearing aid users, followed by older adults with normal hearing, and then middle-aged adults. We also predict a differential pattern of neural plasticity as a function of age/hearing group following executive function training.

## Methods/design

### Design

We will conduct a multi-site, single-blinded, randomized controlled trial involving at-home executive function training over a 12-week period. A total sample of 60 participants will be recruited at each testing site, comprised of 20 middle-aged adults (aged 45–60 years old) with normal hearing, 20 older-adults (aged 65–80 years old) with normal hearing, and 20 older-adults (aged 65–80 years old) with hearing impairment who use hearing aids (with a minimum of 6 months of experience using hearing aids). Participants will be randomized to either an executive function training group or a wait-list control group. The executive function training group will complete the home-based computerized cognitive training program during a 12-week period, while the wait-list control group will not complete any form of cognitive training during the 12 weeks. Participants in the wait-list control group will be offered the cognitive training program at the end of the study. Before and after the 12 week-period, participants in both groups will be asked to complete a series of cognitive, sensory, and mobility tests during two separate in-person sessions.

### Settings

Data collection will be conducted at two separate sites: Montreal (Concordia University), and Toronto (KITE-Toronto Rehabilitation Institute-University Health Network). Participants will be recruited through advertisements displayed at Concordia University’s PERFORM Centre and at the Toronto Rehabilitation Institute, advertisements displayed around the Montreal and Toronto communities (e.g., hospitals, community centres), print/online advertorials targeting our target populations (e.g., Senior Times Montreal), and by contacting participants who previously completed other studies in our labs.

### Participants

Participants will be asked if they were born biologically male or female and an equal number of males and females will be recruited into each group. Hearing loss is known to be more common, more severe, and has an earlier age of onset in males compared to females [48, 49]. A balanced design will therefore allow us to account for the known sex-related differences in hearing loss, in that we will be able to compare performance on our outcome measures across males and females.

### Inclusion criteria

To be enrolled in the study, participants must be aged 45–60 years old and have normal hearing, or 65–80 years old and have either normal hearing or bilateral hearing loss and use hearing aids (with a minimum of 6-months of experience using that set of hearing aids). Hearing ability will be assessed using pure-tone audiometry. An audiometric pure tone threshold average (PTA) will be calculated for each participant across 500 Hz, 1 kHz, 2 kHz, and 4 kHz. Participants will be included in the study if they have a PTA that is equal to or below 25 dB hearing level (HL) in the better ear for the normal-hearing middle-aged and older adults, and above 25 dB HL in both ears for the older adult hearing aid users [50]. While the grades of hearing loss have recently been updated [51], which categorize normal hearing as < 20 dB HL, we have opted to use the previous threshold of ≤ 25 dB HL for comparability with previous studies. All participants must be fluent English speakers and readers (i.e., monolingual or second language acquired early in life). Participants are also required to be physically present at Concordia University’s Loyola Campus in Notre-Dame-de-Grâce, Québec, Canada or the Toronto Rehabilitation Institute, University Centre in Toronto, Ontario, Canada, for a total of four in-person assessment sessions.

### Exclusion criteria

Participants will be excluded from the study if they have a learning disability, attention-deficit hyperactivity disorder, any major psychiatric illnesses (e.g., severe anxiety or depression), any previously diagnosed form of cognitive impairment, or any neurological, cardiovascular, orthopedic, or musculoskeletal conditions that may impede their mobility, concentration, or everyday functioning. Additionally, participants will be administered the Montreal Cognitive Assessment (MoCA) [52], a screening tool for mild cognitive impairment. A score of 26/30 or below on the MoCA may indicate possible mild cognitive impairment (MCI). However, more recent research has shown that a cut-off of 23/30 has better diagnostic accuracy and a lower false positive rate in detecting MCI [53]. As such, all participants must score above or equal to 23/30 on the MoCA, as well as perform within the age-normative range on all other neuropsychological tests (see details below). If a participant’s score on the MoCA falls below 23/30, or they demonstrate below average performance on any other neuropsychological test, they will be excluded from the study. Participants must not engage in any other cognitive training programs for the entire duration of the study and must maintain a consistent physical health lifestyle (i.e., not start a new intensive strength training regime midway through the study). Participants are also required to have normal or corrected-to-normal distance visual acuity and will be excluded if they score above 0.08 on the ETDRS Visual Acuity Chart [54], corresponding to a Snellen acuity equivalent of 20/24 or 6/7. This is an exclusion criterion as our study protocol has some visual requirements that participants need to be able to accomplish (e.g., neuropsychological testing, executive function training). Additionally, research has demonstrated that visual impairment, like hearing loss, is a potentially modifiable risk factor for dementia [55], so we want to control for this possible confound. Lastly, participants exhibiting poor mobility and balance on the Mini-BESTest [56], with a score falling below 16 out of 28, will not be included in the study due to an indication of potential motor difficulties [57].

### Sample size

Sample size calculation is based upon prior intervention studies that utilized the same cognitive training protocol and that reported pre- and post-training changes in our primary outcome measures (e.g., gait or postural variables, auditory working memory) for healthy older adults. Specifically, Pothier et al. [58] used the same executive function training as the current study combined with placebo stretching as one of four training groups and reported a significant Time (pre- vs. post-training) by Training Group interaction (total *n* = 90, *d* = 0.304, actual power = 0.96). For the group receiving the same cognitive training as the current study, a moderate effect of Time on gait speed was found (*n* = 23, *d* = 0.478), with an average increase of 0.13 m/s following training. Additionally, Bruce et al. [46] used a similar cognitive training protocol as the current study in combination with recumbent bicycling (delivered either sequentially or simultaneously), where a significant main effect of Training Group (sequential vs. simultaneous training format) was found for auditory *n*-back accuracy (*n* = 42, *d* = 0.718, actual power = 0.95). The effect size for auditory *n*-back improvement was large, whereas the effect size for gains in mobility was small to moderate. This is consistent with results from Downey et al. [40] where the same cognitive training protocol as the current study was utilized, demonstrating a large effect size for improvements in* n*-back dual-task costs (*g* = -0.83), but a small effect size for gains in walking speed dual-task costs (g =  − 0.11). The recommended (G*Power) sample size to achieve a power of 0.95 at an alpha level of 0.05 for the Training Group x Time interaction for walking speed is 18 per group. Therefore, we aim to recruit 20 participants in each of the three groups (normal-hearing middle-aged adult, normal-hearing older adults, older hearing aid users) to allow for 10% attrition and a balanced design.

## Measures

### Primary outcome measures

The primary outcome measures will include tests of single- and dual-task cognitive, gait, and postural performance in a laboratory environment, including simulated realistic conditions. The outcome measures of interest will include cognitive accuracy and reaction times, temporal gait characteristics, posturography variables, and dual-task cost scores. The older hearing aid users will be required to wear their hearing aids during all testing procedures. Two sets of research assistants will be present at each testing site—one to work with the participant and provide instructions for the tasks, and the other to work with the technical apparatus and ensure that the data are being captured and recorded appropriately.

### PERFORM Protocol at Concordia University in Montréal

*Auditory 2-Back Task.* An auditory 2-back task [41] will be used to measure working memory performance under single- and dual-task conditions. Randomly ordered single digits will be presented at a rate of every two seconds, with each trial lasting 30 s. Single digits (1–10, excluding 7) will be presented through two loudspeakers (Logitech Z623 2.1), at ear-level in height, at a 45-degree angle relative to the listener. Using two hand-held USB response buttons (Black Box ToolKit hand-held USB response button), participants will make a button response to indicate whether the number they are currently hearing is the same or different than the number presented *n* steps previously. Response buttons will be held in both hands, with each connected to a USB response box with a 2-m cable to allow for natural arm swing while walking. Participants will first complete an auditory 1-back task (i.e., indicate whether the number is the same or different than the number presented 1 step previously) in order to individually adjust the volume of the speakers. The decibel level will be continuously reduced until the participant is unable to attain 100 percent accuracy on the 1-back task or until they indicate that listening becomes effortful (i.e., will be asked if they needed to strain in order to understand the digits). Participants will first complete this task while standing and then while walking to ensure audibility with the added background noise of the treadmill. The amplitude of the stimuli will be set for the duration of the experiment at the decibel level from the walking trial where the participant achieved 100 percent accuracy and did not report that listening was effortful. During the 2-back task, participants will use the response buttons to indicate if the number is the same or different than the number presented 2 steps previously. Accuracy and reaction times will be measured. During the single-task 2-back condition, participants will complete the 2-back task while standing, with their feet on the sides of the treadmill. Participants will be required to hold the response buttons during the entire duration of the experiment in order to control for hand positioning but will be allowed to rest their arms on the treadmill railings during single-task 2-back conditions in order to provide postural support.

*Walking Task.* Participants will be required to walk on a treadmill at a self-selected speed at zero percent incline. Specifically, the speed of the treadmill will be gradually increased until the preferred speed is chosen and there are no signs of the participant sweating or being out of breath. The speed of the treadmill will be set at this speed for the duration of the experiment (i.e., will be the same speed across both single-task and dual-task conditions). During single-task conditions, participants must walk on the treadmill at their self-selected speed. During dual-task conditions, participants will simultaneously complete the 2-back task while walking. In order to measure the temporal characteristics of gait (i.e., mean and variability of step time, stride time), two electronic pressure sensors (TeleMyo Direct Transmission System) will be taped to the toe and heel of each shoe sole. Note that the treadmill will be running at the selected speed throughout the entire experiment in order to equate the same level of background noise (i.e., 50 dB SPL) across all trials.

There will be three different trial types (i.e., single-task walking, single-task 2-back, dual-task) and the experiment will follow an ABC-CBA sequence, which will occur two times, for a total of 12 trials. Dual-task costs (DTC; %) will be calculated for each of the walking and 2-back tasks: [(single-task – dual-task) / single task * 100]. DTC change scores will be calculated by subtracting the post-training DTCs from the pre-training DTCs.

### StreetLab Protocol at UHN/KITE in Toronto

**Fig. 1:**
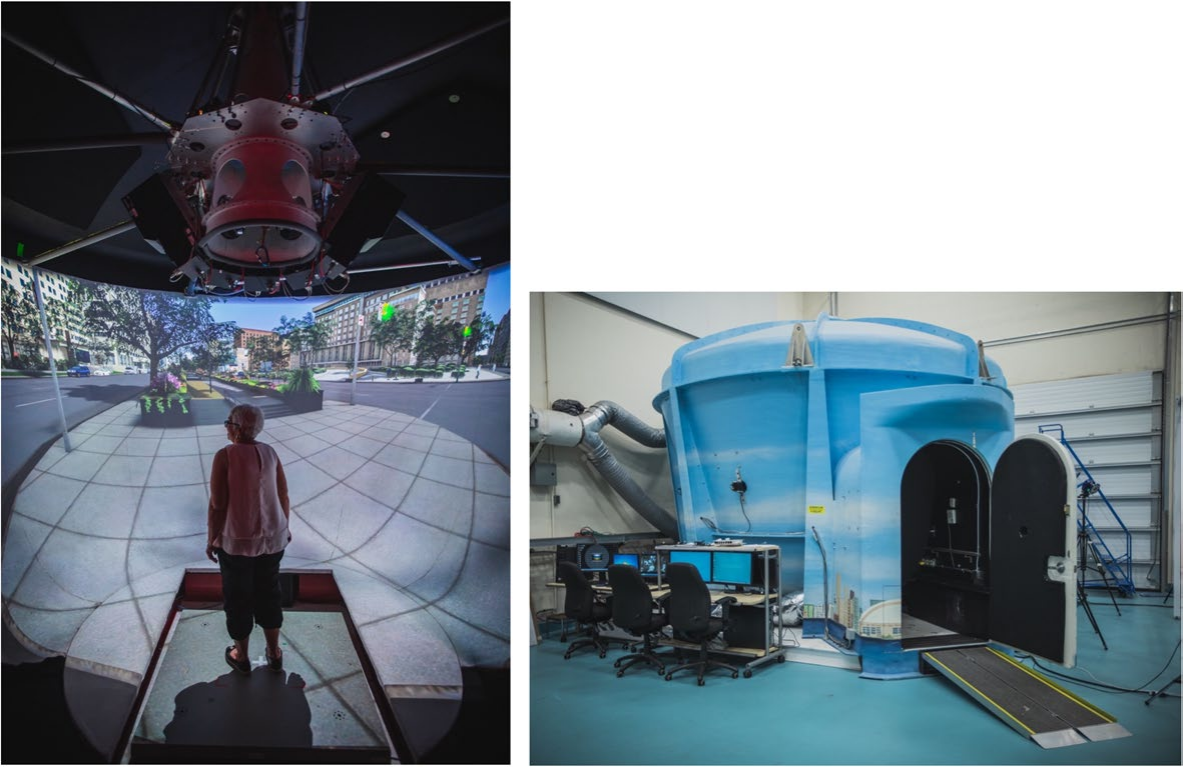
StreetLab *Note.* Participants will either stand in the centre of the force platform with a safety harness or be seated. Under dual-task conditions, participants will be standing while concurrently performing either the auditory 2-back task or the coordinated response measures task

 Two dual-task paradigms will be conducted in StreetLab, a fully immersive, projection-based, VR simulator used to simulate realistic and challenging conditions (Fig. 1). StreetLab has a 240° horizontal by + 15° to -90° vertical field-of-view curved projection screen extending from the floor to ceiling. The virtual environment used in this study will depict a large urban 6-lane intersection street-crossing in Toronto. StreetLab is outfitted with an AMTI (Advanced Mechanical Technology, Inc., Watertown, MA) BP12001200–2000 strain gage force plate that measures ground reaction forces. There is a surround sound system with seven speakers (Meyersound MP-4XP; Meyersound Laboratories, Inc., Berkeley, CA) spatially distributed behind the projection screen, at approximately the height of a participant’s head when they are standing on the force plate positioned at 0**°** azimuth across a horizontal plane at ± 28° (right front and left front), ± 90° (right side and left side), and ± 127° (right rear and left rear). One subwoofer (Meyersound MP-10XP) is located in the floor under the centre speaker. All speakers are 2.14 m in depth from the participant when standing on the force plate (for more details of the acoustic properties of StreetLab, refer to [61]). Ambient traffic noise (e.g., vehicle traffic, bird noises) will be included to more closely simulate real-world acoustical conditions. The vehicle traffic will include moderate traffic density with approximately 10 cars appearing in the visual scene every 30 s. Participants will complete two dual-task paradigms that largely differ based on the two different cognitive auditory tasks to be performed, including 1) auditory 2-back task and 2) Coordinated Response Measures (CRM) task. The other primary difference from the Concordia site protocol is that instead of measuring walking as the primary mobility-related outcome measure, we will be measuring standing balance under different levels of cognitive and postural complexity.

### Auditory 2-back paradigm

*2-Back Task:* The same auditory 2-back task as the Concordia site [41] will be used; however, trials will last 60 s instead of 30 s in order to capture a sufficient amount of reliable posturography data [25]. The amplitude of the loudspeakers will be individually adjusted using an auditory 1-back task to ensure optimal audibility and comfort across participants. Specifically, participants will hear 15 untimed, single digits where they will have to repeat the digit they heard out loud to the researcher immediately after the digit is presented. In order to participate in the study, participants must attain at least 70% accuracy or higher. If the amplitude is uncomfortably loud, it will be reduced (ensuring at least 70% accuracy) and the amplitude will then be set at this level for the remainder of the experiment. Accuracy and reaction time will be measured using a handheld gaming controller (Forty4 Wireless Gaming Controller). During the main experimental task, participants will make a button press indicating whether the number they are currently hearing is the same or different than the number presented *n* steps previously (i.e., 1-step previously during the practice trials, or 2-steps previously for the 2-back task). During single-task conditions, the participants will complete the 2-back while sitting on a chair on top of the force plate within StreetLab at the same approximate eye height as standing.

*Postural Task.* Static balance will be measured by having participants stand with their feet shoulder-width apart on a force platform. In order to control for differences in hand positions across the single-task standing and dual-task trials (since participants use handheld joysticks during the 2-back task), participants will be required to hold the joystick in the same position as the dual-task trials (but will not need to make any button responses). In order to manipulate postural load, participants will complete the standing balance task with their eyes open and eyes closed. Postural measures will include spatial (centre of pressure path length; cm), temporal (velocity; cm/s) and variability (root means square, standard deviation) measures in the anterior–posterior (front and back) and medial–lateral (side-to-side) orientations.

Overall, there will be a total of six trial types: 1) single-task 2-back eyes open, 2) single-task 2-back eyes closed, 3) single-task standing eyes open, 4) single-task standing eyes closed, 5) dual-task eyes open, 6) dual-task eyes closed. Participants will complete all trial types in an ABC-CBA sequence with the three different conditions (A: single-task standing, B: single-task 2-back, and C: dual-task). This will occur two times, once for the eyes open condition, and once for the eyes closed condition. The order of trials (i.e., eyes open vs. eyes closed) will be counter-balanced across participants. DTCs (percentage) will be calculated for both the standing and 2-back tasks: [(single-task – dual-task) / single task * 100]. DTC change scores will be calculated by subtracting the post-training DTCs from the pre-training DTCs.

### CRM Paradigm

*CRM Task*: The CRM is an auditory, multi-talker task [59], which we have adapted for use in dual-task experiments in a VR environment [19, 60]. Participants will hear two simultaneously presented, but spatially distributed sentences. Each sentence will be composed of the following structure *“Ready (callsign) go to (colour) (number) now”.* A combination of 8 callsigns (Charlie, Ringo, Laker, Tiger, Arrow, Baron, Eagle, Hopper), 4 colours (red, green, white and blue) and 7 numbers (1–8 without 7) are used to compose each sentence. On each trial, a target callsign will be visually presented in text at the centre of the projection screen (e.g., Charlie). The size of a single character will be roughly 8 cm x 8 cm and the distance between the participant and the text will be roughly 2.1 m. Subsequently, two sentences will be simultaneously presented from the front speaker (0 degrees) and left speaker (- 90 degrees), one of which will contain the target word (e.g., the sentence “*Ready Charlie go to White 2 now*)”. Participants will be asked to verbally repeat the colour (i.e., white) and the number (i.e., 2) associated with the target callsign sentence. The researcher will enter the participants’ responses on a tablet (Samsung SM-T510). If the participant accurately repeats the colour and number, the trial is coded as correct and they receive feedback on the screen (“correct”) and if either the colour, the number, or both are incorrectly reported “incorrect” is presented on the screen. For each condition (see below), five listening trials/sentences will be presented within a 60 s standing trial, which will be repeated two times per condition (10 total listening trials). During single-task conditions, the participants will complete the CRM while sitting on a chair on top of the force plate within StreetLab at the same approximate eye height as standing.

In order to manipulate attentional load and listening difficulty, we will include a block of trials in which the location of the target word will be certain 100% of the time (i.e., participants will be told the target word will always be presented at 0 degrees; termed 100% trials; lower cognitive load) and a block of trials in which the location of the target word will be uncertain, presented from the centre 60% of the time and from the left 40% of the time (i.e., participants will be told that the target word will be coming from the middle 60% of the time and from the left 40% of the time; termed 60% trials; higher cognitive load). Participants will complete these 100% and 60% trial types in single and dual-task conditions.

*Postural Task*. Static balance will be measured on a force platform in a semi-tandem stance, with one foot slightly in front of the other. Postural measures will include spatial (centre of pressure path length; cm), temporal (velocity; cm/s) and variability (root means square, standard deviation) measures in the anterior–posterior (front and back) and medial–lateral (side-to-side) orientations.

Overall, there will be a total of 5 different trial types: 1) single-task CRM (100%/low cognitive load), 2) single-task semi-tandem standing, 3) dual-task (60%/high cognitive load), 4) single-task CRM (60%/high cognitive load), 5) dual-task (100%/low cognitive load). Participants will complete all trial types in this order and will then repeat the trials in the reverse order, for a total of 10 trials. DTCs (percentage) will be calculated for both the standing and CRM tasks: [(single-task – dual-task) / single task * 100]. DTC change scores will be calculated by subtracting the post-training DTCs from the pre-training DTCs.

### Secondary outcome measures

A set of secondary outcome measures will evaluate *cognitive functioning* using a series of neuropsychological tests. The Coding subtest of the Weschler Adult Intelligent Scale (WAIS-IV) will be used as a measure of visual-motor processing speed, the Digit Span subtest—forward condition will be used to measure short term memory and the Digit Span subtest- backwards condition and Letter-Number-Sequencing subtest will be used to assess auditory working memory [62]. The Trail Making Test A & B will serve to evaluate processing speed and task-switching abilities [63]. The Color Word Inference Test will be used to measure processing speed, inhibition and task-switching abilities [64]. Finally, the Rey Auditory Verbal Learning Test (RAVLT) will measure participants’ learning and retention of verbal information [65]. Different versions of the RAVLT, consisting of different word lists, will be administered at baseline and post-training in order to reduce possible practice effects. In order to determine whether participants fall within the age-normative range and can be included in the study, performance will be compared to normative data taken from the following sources: Wechsler et al., [62] for the WAIS-IV measures, Delis et al., [64] for the Color Word Interference Test, Tombaugh et al., [66] for the Trail Making Test, and Schmidt [67] for the RAVLT.

Secondary measures will also assess *sensory functioning* using a selection of visual and auditory tasks. For visual outcomes, the Pelli-Robson chart [68] will be used to assess contrast sensitivity for reading letters. For auditory outcomes, the Canadian Digit Triplets Test (CDTT) [69] will measure participants’ ability to identify digits in the presence of competing noise (i.e., their speech-in-noise perception threshold). Participants will be presented with digit triplets unaided via headphones (Telephonics TDH-39P Audiometer Headset) and asked to enter their responses into a numerical keypad (Peripad-202 HW, Perixx Computer). Pure-tone audiometry will also be conducted using a SHOEBOX Audiometer to assess participants’ hearing acuity [70]. Participants will be presented tones via headphones (RadioEar DD450) at 250 Hz, 500 Hz, 1 kHz, 2 kHz, 4 kHz, and 8 kHz at varying decibel levels in each ear. Participants will be instructed to indicate whether they can hear the tone or not by making a button response on a tablet (iPad; Apple Inc.). Performance from tones presented at the 500 Hz, 1 kHz, 2 kHz, and 4 kHz frequencies will be averaged together to create an individual PTA for the left and right ear.

A shortened version of the Balance Evaluation Systems Test called the Mini-BESTest [56] will be used to assess *motor functioning*. Postural control across four different balance control systems will be quantifiably measured, including: anticipatory transition (e.g., going from sitting to standing), reactive postural control (e.g., leaning outside one’s centre of pressure and compensating for a loss of balance), sensory orientation (e.g., balancing with eyes closed or on a compliant surface), and dynamic gait (e.g., walking while turning head, changing speed, or stepping over obstacles).

A set of questionnaires will also be given to participants to complete online using Qualtrics survey software (Qualtrics, Provo, Utah) in order to assess *subjective functioning* across cognitive, hearing, and mobility domains. Specifically, within the domain of cognition, the Frequency of Forgetting Questionnaire (FFQ) will be used to assess subjective memory abilities and how frequently one forgets things in everyday life [71]. In the domain of hearing, the Listening Self-Efficacy Questionnaire (LSEQ) will be used to assess one’s self-efficacy or confidence in understanding speech in a variety of listening situations [72], and the Hearing Handicap Inventory Screening Questionnaire for the Elderly (HHIE-S) will be given to assess self-reported experiences of hearing difficulties in everyday life [73]. Within the domain of motor functioning, the Activities-Specific Balance Confidence (ABC) Scale will be used to determine participants’ balance efficacy [74]. Additionally, participants will be asked to complete the Everyday Activity Questionnaire (EAQ), which samples a broad range of activities relevant to older adults’ lives (maintenance of self and property, social, leisure, religious, and creative activities) [75]. Lastly, the Social Disengagement Inventory (SDI) will be used to measure how participants interact and engage with their social environment [76].

Brain activity will be measured (Pre- and Post-Training) using functional near infrared spectroscopy (fNIRS) at Concordia’s PERFORM Centre. The working brain is in constant need of supply of glucose and oxygen for efficient functioning. As such, levels of oxygenated hemoglobin (HbO) and deoxygenated hemoglobin (HbR) in the brain can be used as neurobiological indicators of cortical activation. Increased brain activation leads to increased blood volume and blood flow, resulting in an increase in HbO and a decrease in HbR. Conversely, decreased brain activation leads to decreased blood volume and blood flow, resulting in a decrease in HbO and an increase in HbR. Functional near infrared spectroscopy provides a non-invasive measure of HbO and HbR fluctuations in the cerebral cortex with high temporal resolution [77]. A mobile (wireless/Bluetooth) fNIRS device will be used to allow participants to freely complete the experimental task while walking (Artinis Brite MKIII, Netherlands). Light optode placement will follow a 24-channel frontal template provided by the analysis software OxySoft (Version 3.3.30). Data pre-processing involving the visual inspection of motion artifacts, detection of bad channels, Modified Beer-Lambert Law (MBLL) conversion, and band pass filtering (0.005 Hz-0.1 Hz), will be conducted using MATLAB (Version R2021b) and the open-source application Brainstorm [78]. Additionally, all trials will be normalized to baseline, which will be taken during a quiet standing condition prior to each trial. A single HbO activation average will be computed for each participant for each trial type (i.e., single-task walking, single-task 2-back, and dual-task). HbO change scores will then be derived to compare Pre- and Post-Training cortical activation levels.

### Study procedure

**Fig. 2:**
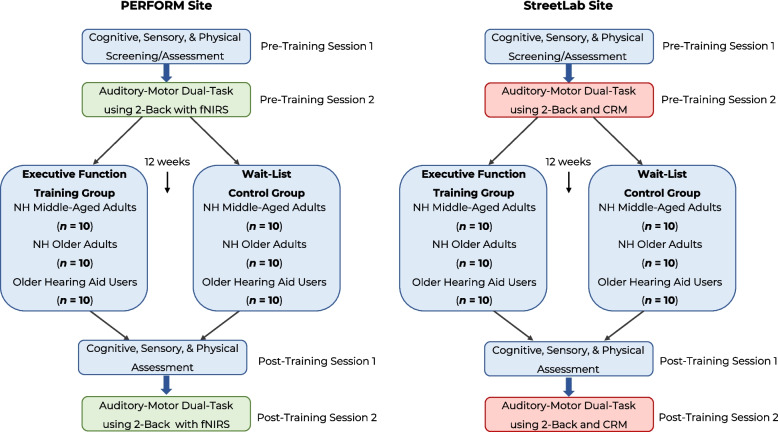
Flowchart of measures and timeline of our multi-site, single-blinded, randomized controlled trial *Note*. Each site will be comprised of a sample of 20 middle-aged adults and 20 older adults with normal hearing (NH), as well as 20 older adults with age-related hearing loss who use hearing aids. An equal number of males and females will be recruited into each training group within each of the age/hearing groups. Both the executive function training group and wait-list control group will participate in Pre- and Post-Training assessments. During the 12-week intervention phase, the executive function training group will complete an at-home cognitive training program three times a week (30 minutes/session). The wait-list control group will not complete any form of cognitive training during the duration of the experiment, but will be offered the materials at the end of the study

Prior to any in-person visit, a telephone screening will be conducted to determine a participant’s initial eligibility to participate in the study (i.e., will gather demographic information and a basic medical history, as well as the participant’s comfort level using a computer or tablet). Eligible participants will be asked to come into Concordia University’s Loyola Campus (Montreal location) or KITE-Toronto Rehabilitation Institute, University Health Network (University Centre location) to provide written consent, be further screened using a more in-depth health history interview, and be assessed on various cognitive and sensory measures, including pure-tone audiometry, CDTT, MoCA, Pelli-Robson Contrast Sensitivity, RAVLT, Coding, Digit Span, Trail Making Test A & B, Letter-Number-Sequencing, and Color Word Inference Test (Pre-Training Session 1). Testing will take place in a quiet, well-illuminated room. If deemed eligible, participants will be asked to come into Concordia University’s PERFORM Centre (Montreal location) or KITE-Toronto Rehabilitation Institute, University Health Network (University Centre) to assess our primary outcome measure (i.e., cognitive-motor dual-tasking) and other measures including the ETDRS and Mini-BESTest (Pre-Training Session 2). Participants will also be asked to complete a series of questionnaires online between Pre-Training Sessions 1 and 2 (i.e., LSEQ, ABC, EAQ, FFQ, HHIE-S, SDI). Participants will then be randomized into either the executive function training group or the wait-list control group. The executive function training group will receive a virtual tutorial session on how to use the at-home cognitive training program. Participants will then undergo either 12 weeks of cognitive training or will continue their life as usual (wait-list control group). Both groups will be called once a week to keep track of any changes in lifestyle, stress, energy levels or any other important life events. The executive function training group will also be asked about their training that week (e.g., if they are having any difficulty with the training or need further clarification, if they are noticing any progress/improvements, if they are experiencing any technical issues, if they missed a session). Following the 12-week period, all participants will be invited back to complete the same sensory, cognitive, and motor assessments that they completed at baseline (Post-Training Session 1 and Post-Training Session 2). Participants will also complete the LSEQ, ABC, and FFQ questionnaires again to measure changes in subjective hearing, mobility, and cognition, respectively. An overview of the study procedure across each testing site is shown in Fig. 2.

### Randomization

Upon completion of the screening and baseline assessments, participants will be randomized to either the executive function training or wait-list control group. Randomization of participants will be completed within each of the three groups (i.e., middle-aged, older adults, hearing aid users) using a computerized random number generator. Final cohorts will be semi-randomized in order to attain an equal number of participants in the training and control groups and to balance the number of males and females within each age/hearing group.

### Blinding

Participants will be made aware of the two groups in which they could be randomized to and will know which of the two groups they have been assigned to following the baseline assessments. Efforts will be made so that the evaluators conducting the Pre- and Post-Training assessments will be blind to the participants’ randomization status. For example, the research personnel responsible for calling participants during the 12-week period will be different than the research personnel completing the post-training evaluations.

### At-home executive function training group

After the Pre-Training phase, participants randomized to the executive function training group will complete an at-home computerized cognitive training program three times a week for a total of 12 weeks (each session lasting 30 min). The training protocol will involve a custom-written program that has been used in previous research studies assessing cognitive and gait outcomes, but was previously administered in a controlled laboratory environment [39–41, 43]. Specifically, the training is comprised of three distinct modules (Stroop, dual-task, *n*-back), which aim to improve different aspects of executive functioning such as inhibition and task switching-abilities, divided attention, and working memory, respectively. Participants will be encouraged to evenly disperse the training throughout the week (i.e., 48 h between sessions). However, participants have the flexibility to complete the sessions according to their own schedule. Each training session (30 min) is comprised of two different modules (each 15 min). Participants may take a break between modules, but not during a module. The computerized program can be completed on either a laptop, desktop, or tablet, but should be held consistent throughout the 12-week duration of the training. Participants will also be instructed to work as fast as they can without making any mistakes, as both reaction time (milliseconds) and accuracy will be recorded for all three tasks. After each session, a performance feedback graph will be presented to provide encouragement and allow participants to track their progress.

The Stroop module aims to improve inhibition and task-switching using four separate conditions (familiarization, reading, inhibition, and switching) presented at different points throughout the training period. Participants will be required to press the appropriate letter key on their keyboard or press the appropriate button on their tablet, based on the condition presented. In the familiarization condition, a single letter will be presented on the screen. In the reading condition, asterisks will form a letter on the screen. In the inhibition condition, smaller letters will make up a larger letter (e.g., copies of small letters “L” to form a larger “H”), and participants will need to inhibit the automatic response of indicating what the smaller letter is, and instead indicate what larger letter is formed. In the switching condition, participants will need to alternate between reporting the smaller letters presented, or the larger letters that are formed, depending on the goal as indicated by whether a white frame is surrounding the group of letters or not.

The visual *n*-back module targets the updating and maintenance of information in working memory. Participants will be instructed to make a keyboard response or button press on a tablet indicating whether the letter they see is the same or different from the one presented one previously (1-back), two previously (2-back), or three previously (3-back). The more challenging 2-back and 3-back trials will make up a larger proportion of the trial type distribution as the training progresses in order to enhance the difficulty level. Of note, the modality of the *n*-back in the cognitive training differs from the modality used in the dual-task experiment (i.e., visual during training versus auditory during the dual-task experiment).

The dual-task module aims to improve divided attention. Participants will complete a visual discrimination task whereby they will need to make a response to one type of image (single-task; i.e., modes of transportation or fruits;) or two types of images concurrently (dual-task; i.e., one image reflecting a mode of transportation and another image of a fruit). Participants will complete pure blocks of only single-task trials (single-pure trials) or only dual-task trials (dual-pure trials) or will complete mixed blocks that randomly involve one (single-mixed trials) or both tasks (dual-mixed trials). For the dual-task trials, participants will be instructed to respond to both stimuli equally. However, as the training progresses, participants will need to prioritize one hand over the other to increase the level of difficulty (i.e., in the dual-task trials when two images are presented, participants will be asked to make a response using their left or right hand first before making a response with the other hand).

### Waitlist control group

Participants randomized to the wait-list control group will complete both the Pre- and Post-Training assessments but will not partake in any form of cognitive training during the 12-week period. Weekly phone calls will ensure that participants are maintaining a consistent lifestyle and that they are not starting any other cognitive training programs. The weekly phone calls will also allow for a similar level of social interaction amongst the research personnel and the participants across the wait-list control and training groups in order to control for this possible confound. Upon completing the study, the wait-list control group will be offered the materials for the at-home executive function training program, and researchers will be available to offer assistance if needed.

### Data analysis

#### Planned analyses

All data analyses will be completed using IBM SPSS Statistical Software and/or R. Data will be screened and corrected for normality, outliers, and missing values. Descriptive statistics (e.g., means and standard deviations for continuous variables; frequencies and percentages for categorical variables) will be presented for the demographic and baseline characteristics. Chi square tests (for categorical variables) or One-Way ANOVAs (for continuous variables) will be used to determine if there are any differences between groups at baseline (i.e., middle-aged vs. older adults vs hearing aid users; training vs. control groups).

In order to evaluate our primary hypothesis (i.e., that executive function training will lead to greater improvements in dual-task performance compared to a wait-list control group), separate 3 × 2 × 2 Mixed Factorial ANOVAs will be conducted for each of the primary outcome measures (i.e., 2-back accuracy and reaction times, CRM accuracy, temporal gait parameters, posturography variables, DTC scores), whereby the Between-Subjects factors will include Age/Hearing Group (middle-aged, older adult, hearing aid users) and Training Group (cognitive training, control), the Within-Subject factor will include Time (pre- vs. post-evaluations), and Covariates will include sex and education. Observation of a statistically significant Time by Training Group interaction in any of the primary outcome measures will be considered preliminary evidence for training efficacy. In order to examine our second and third hypotheses (i.e., that largest improvements in dual-task performance and greatest augmentation of brain activity will be found in older hearing aid users, followed by normally-hearing older adults, and then middle-aged adults) separate One-Way ANOVAs will be used on the dual-task and HbO change scores within the cognitive training group, whereby the Between Subjects factor will include Age/Hearing Group (middle-aged, older adult, hearing aid users). Observation of a statistically significant main effect of Group will be followed up by post hoc pairwise analyses with Bonferroni corrections in order to determine whether our hypotheses are supported.

Linear mixed effects models will also be fitted for each of our outcome measures, particularly if there is a large amount of missing data or if there is an unequal amount of variance across groups. Specifically, Fixed effects will include Age/Hearing Group (middle-aged, older adult, hearing aid users), Training Group (executive function training, control), Sex (male/female), and Time (Pre- vs. Post-Training), Random effects will include the individual participants, and Covariates will include education.

Lastly, in order to elucidate whether brain activity reflects neural compensation or inefficiency, correlations between dual-task performance and HbO levels will be conducted at baseline and after 12 weeks. All statistical tests will be two-tailed, and a *p*-value of less than 0.05 will be considered to indicate statistical significance. Effect sizes will be calculated using Hedges’ *g.*

### Ethical considerations

Each intervention site obtained approval by their corresponding Research Ethics Board prior to initiating any study-related activities, including Concordia University’s Human Research Ethics Committee (Certificate #30,011,799) and KITE-Toronto Rehabilitation Institute-University Health Network (REB#19–5857).

## Discussion

In this multi-site single-blinded randomized controlled trial, we aim to establish the efficacy of an at-home cognitive training program aimed at improving cognitive and motor functioning under laboratory and simulated real-world conditions in normal-hearing middle aged and older adults and older hearing aid users. We hypothesize that executive function training will lead to greater improvements in dual-task performance compared to a wait-list control group. Moreover, we hypothesize that executive function training will lead to the largest dual-task improvements in older hearing aid users, followed by normal-hearing older adults, and then middle-aged adults. Lastly, we predict a differential pattern of neural plasticity as a function of age/hearing group following executive function training.

Computerized executive function training has been shown to improve cognition and mobility in normal-hearing older adults [38–45]. Preliminary evidence also suggests that combined exercise and cognitive training can improve dual-task performance in older adults with hearing loss [46, 47]. However, further research is needed to investigate the effect of executive function training (in isolation rather than combined with exercise) on cognition and mobility in older adults with hearing loss. Additionally, as telehealth has been increasingly utilized since the COVID-19 pandemic, it is paramount to explore whether similar results remain when cognitive training is completed remotely, rather than in a laboratory setting.

Our study aims to fill these gaps in knowledge and will be the first of its kind to examine the effect of an at-home executive function training program on complex auditory and motor performance in normal-hearing middle-aged and older adults and older hearing aid users. One advantage of our study protocol includes the targeted population given that hearing loss is associated with an increased risk for falls and dementia, which may be modified through hearing aids and cognitive training. We will also recruit normal-hearing middle-aged and older adults in order to examine differences in cognition and mobility that can occur with age and changes in hearing acuity.

Another strength of the study protocol is the use of ecologically valid measurement techniques, including subjective and objective measures of hearing, cognition, and mobility, as well as an immersive, multisensory VR environment to assess realistic sensory-cognitive-motor challenges. Our previous research assessing dual-task performance in a simulated street crossing environment showed that older adults tended to prioritize posture over cognitive performance (e.g., reduced gait variability at the cost of word recognition accuracy) [19, 28]. As such, our study will add to this growing literature and extend previous research using traditional laboratory-based experiments.

A final strength of the protocol is the use of portable fNIRS to examine brain activity in the prefrontal cortex during dual-task walking before and after training. Currently, there are inconsistent findings with regard to the effect of dual-tasking on brain activity across younger and older adults, with some researchers showing a bilateral upregulation in the prefrontal cortex in older adults compared to younger adults [79, 80], others showing comparable brain activity [81], and others showing greater activity in younger adults compared to older adults [82]. It also remains unclear how brain activity during dual-tasking changes following cognitive training. Scaffolding theories propose that training may increase compensatory mechanisms in response to declining brain structure with age via an up-regulation of frontal brain regions [83]. However, the effect of cognitive training on brain activity during dual-tasking in older adults with hearing loss has yet to be examined. We therefore hope that our study will elucidate some of these inconsistencies and contribute to the growing knowledge on mobile brain imaging.

In conclusion, this research will help establish the efficacy of an at-home cognitive training program in improving cognitive and motor functioning in older adults with hearing loss. Given the association between hearing loss and dementia and falls risk, the results of this study may have direct implications for older adults, in terms of improving quality of life and level of autonomy, as well as potentially reducing healthcare costs. Indeed, dementia and falls cause $10.4 and $2 billion a year in healthcare costs, respectively [84, 85]. From a basic science perspective, this research will also contribute to our understanding of how brain activity differs amongst normal-hearing middle-aged and older adults, and older hearing aid users, as well as how brain activity changes in response to cognitive training. These findings will elucidate whether brain activity reflects a compensatory mechanism for declining brain structure due to age and hearing loss, or whether it is a marker of neural inefficiency that can be improved through cognitive training.

## Data Availability

Not applicable as recruitment is still ongoing.

## Abbreviations

AI: Artificial intelligence
VR: Virtual reality
PTA: Pure tone average
MoCA: Montreal Cognitive Assessment
MCI: Mild cognitive impairment
CRM: Coordinated response measures
WAIS-IV: Weschler Adult Intelligent Scale 4Th Edition
RAVLT: Rey Auditory Verbal Learning Test
CDTT: Canadian Digit Triplets Test
FFQ: Frequency of Forgetting Questionnaire
LSEQ: Listening Self-Efficacy Questionnaire
HHIE-S: Hearing Handicap Inventory for Elderly Screening
ABC: Activities-Specific Balance Confidence
EAQ: Everyday Activity Questionnaire
SDI: Social Disengagement Inventory

## References

[1] Lin FR, Thorpe R, Gordon-Salant S, Ferrucci L (2011). Hearing loss prevalence and risk factors among older adults in the United States. J Gerontol A Biol Sci Med Sci.

[2] Mick PT, Hämäläinen A, Kolisang L, Pichora-Fuller MK, Phillips N, Guthrie D, Wittich W (2021). The prevalence of hearing, vision, and dual sensory loss in older Canadians: An analysis of data from the Canadian Longitudinal Study on Aging. Can J Aging..

[3] Lin FR, Yaffe K, Xia J, Xue QL (2013). Harris TB, Purchase-Helzner E, Satterfield S, Ayonayon HN, Ferrucci L, Simonsick EM, Health ABC Study Group FT. Hearing loss and cognitive decline in older adults. JAMA Intern Med.

[4] Lin FR, Metter EJ, O’Brien RJ, Resnick SM, Zonderman AB, Ferrucci L (2011). Hearing loss and incident dementia. Arch Neurol.

[5] Livingston G, Sommerlad A, Orgeta V, Costafreda SG, Huntley J, Ames D, Ballard C, Banerjee S, Burns A, Cohen-Mansfield J, Cooper C (2017). Dementia prevention, intervention, and care. Lancet.

[6] Livingston G, Huntley J, Sommerlad A, Ames D, Ballard C, Banerjee S, Brayne C, Burns A, Cohen-Mansfield J, Cooper C, Costafreda SG (2020). Dementia prevention, intervention, and care: 2020 report of the Lancet Commission. Lancet.

[7] Loughrey DG, Kelly ME, Kelley GA, Brennan S, Lawlor BA (2018). Association of age-related hearing loss with cognitive function, cognitive impairment, and dementia: a systematic review and meta-analysis. JAMA Otolaryngol Head Neck Surg.

[8] Kamil RJ, Betz J, Powers BB, Pratt S, Kritchevsky S, Ayonayon HN, Harris TB, Helzner E, Deal JA, Martin K, Peterson M (2016). Association of hearing impairment with incident frailty and falls in older adults. J Aging Health.

[9] Lin FR, Ferrucci L (2012). Hearing loss and falls among older adults in the United States. Arch Intern Med.

[10] Campos J, Ramkhalawansingh R, Pichora-Fuller MK (2018). Hearing, self-motion perception, mobility, and aging. Hear Res.

[11] Li L, Simonsick EM, Ferrucci L, Lin FR (2013). Hearing loss and gait speed among older adults in the United States. Gait Posture.

[12] Baltes PB, Lindenberger U (1997). Emergence of a powerful connection between sensory and cognitive functions across the adult life span: a new window to the study of cognitive aging?. Psychol Aging.

[13] Lindenberger U, Baltes PB (1994). Sensory functioning and intelligence in old age: a strong connection. Psychol Aging.

[14] Pichora-Fuller MK (2003). Cognitive aging and auditory information processing. Int J Audiol.

[15] Schneider BA, Pichora-Fuller MK, Craik FI, Salthouse TA (2000). Implications of perceptual deterioration for cognitive aging research. The handbook of aging and cognition.

[16] Li KZH, Bherer L, Mirelman A, Maidan I, Hausdorff JM (2018). Cognitive involvement in balance, gait and dual-tasking in aging: a focused review from a neuroscience of aging perspective. Front Neurol.

[17] Peelle JE (2018). Listening effort: how the cognitive consequences of acoustic challenge are reflected in brain and behavior. Ear Hear.

[18] Bruce H, Aponte D, St-Onge N, Phillips N, Gagné JP, Li KZ (2019). The effects of age and hearing loss on dual-task balance and listening. J Gerontol B Psychol Sci Soc Sci.

[19] Lau ST, Pichora-Fuller MK, Li KZ, Singh G, Campos JL (2016). Effects of hearing loss on dual-task performance in an audiovisual virtual reality simulation of listening while walking. J Am Acad Audiol.

[20] Davis MH, Johnsrude IS (2003). Hierarchical processing in spoken language comprehension. J Neurosci.

[21] Wong PC, Jin JX, Gunasekera GM, Abel R, Lee ER, Dhar S (2009). Aging and cortical mechanisms of speech perception in noise. Neuropsychologia.

[22] Campbell J, Sharma A (2013). Compensatory changes in cortical resource allocation in adults with hearing loss. Front Syst Neurosci.

[23] Erb J, Obleser J (2013). Upregulation of cognitive control networks in older adults’ speech comprehension. Front Syst Neurosci.

[24] Rosemann S, Thiel CM (2018). Audio-visual speech processing in age-related hearing loss: Stronger integration and increased frontal lobe recruitment. Neuroimage.

[25] Carpenter MG, Campos JL (2020). The effects of hearing loss on balance: a critical review. Ear Hear.

[26] Keidser G, Naylor G, Brungart DS, Caduff A, Campos J, Carlile S, Carpenter MG, Grimm G, Hohmann V, Holube I, Launer S (2020). The quest for ecological validity in hearing science: What it is, why it matters, and how to advance it. Ear Hear.

[27] Meilinger T, Knauff M, Bülthoff HH (2008). Working memory in wayfinding - a dual task experiment in a virtual city. Cogn Sci.

[28] Nieborowska V, Lau ST, Campos J, Pichora-Fuller MK, Novak A, Li KZ (2018). Effects of age on dual-task walking while listening. J Mot Behav.

[29] Neider MB, Gaspar JG, McCarley JS, Crowell JA, Kaczmarski H, Kramer AF (2011). Walking and talking: dual-task effects on street crossing behavior in older adults. Psychol Aging.

[30] McGarrigle R, Munro KJ, Dawes P, Stewart AJ, Moore DR, Barry JG, Amitay S (2014). Listening effort and fatigue: What exactly are we measuring? A British Society of Audiology Cognition in Hearing Special Interest Group ‘white paper’. Int J Audiol.

[31] Humes LE, Rogers SE, Quigley TM, Main AK, Kinney DL, Herring C (2017). The effects of service-delivery model and purchase price on hearing-aid outcomes in older adults: A randomized double-blind placebo-controlled clinical trial. Am J Audiol.

[32] Humes LE, Kinney DL, Main AK, Rogers SE (2019). A follow-up clinical trial evaluating the consumer-decides service delivery model. Am J Audiol.

[33] McArdle R, Chisolm TH, Abrams HB, Wilson RH, Doyle PJ (2005). The WHO-DAS II: measuring outcomes of hearing aid intervention for adults. Trends Amplif.

[34] Sanders ME, Kant E, Smit AL, Stegeman I (2021). The effect of hearing aids on cognitive function: a systematic review. PLoS ONE.

[35] Sanchez VA, Arnold ML, Reed NS, Oree PH, Matthews CR, Eddins AC, Lin FR, Chisolm TH (2020). The Hearing Intervention for the Aging and Cognitive Health Evaluation in Elders randomized control trial: Manualization and feasibility study. Ear Hear.

[36] Borsetto D, Corazzi V, Franchella S, Bianchini C, Pelucchi S, Obholzer R, Soulby AJ, Amin N, Ciorba A (2021). The influence of hearing aids on balance control: a systematic review. Audiol Neurootol.

[37] Lawrence BJ, Jayakody DM, Henshaw H, Ferguson MA, Eikelboom RH, Loftus AM, Friedland PL (2018). Auditory and cognitive training for cognition in adults with hearing loss: A systematic review and meta-analysis. Trends Hear.

[38] Bherer L, Gagnon C, Langeard A, Lussier M, Desjardins-Crépeau L, Berryman N, Bosquet L, Vu TT, Fraser S, Li KZ, Kramer AF (2021). Synergistic effects of cognitive training and physical exercise on dual-task performance in older adults. J Gerontol B Psychol Sci Soc Sci.

[39] Desjardins-Crépeau L, Berryman N, Fraser SA, Vu TT, Kergoat MJ, Li KZ, Bosquet L, Bherer L (2016). Effects of combined physical and cognitive training on fitness and neuropsychological outcomes in healthy older adults. Clin Interv Aging.

[40] Downey R, Bherer L, Pothier K, Vrinceanu T, Intzandt B, Berryman N, Lussier M, Vincent T, Karelis AD, Nigam A, Vu TT, Bosquet L, Li KZH (2022). Multiple routes to help you roam: A comparison of training interventions to improve cognitive-motor dual-tasking in healthy older adults. Front Aging Neurosci.

[41] Fraser SA, Li KZ, Berryman N, Desjardins-Crépeau L, Lussier M, Vadaga K, Lehr L, Minh Vu TT, Bosquet L, Bherer L (2017). Does combined physical and cognitive training improve dual-task balance and gait outcomes in sedentary older adults?. Front Hum Neurosci.

[42] Li KZ, Roudaia E, Lussier M, Bherer L, Leroux A, McKinley PA (2010). Benefits of cognitive dual-task training on balance performance in healthy older adults. J Gerontol A Biol Sci Med Sci.

[43] Pothier K, Vrinceanu T, Intzandt B, Bosquet L, Karelis AD, Lussier M, Vu TM, Nigam A, Li KZ, Berryman N, Bherer L (2021). A comparison of physical exercise and cognitive training interventions to improve determinants of functional mobility in healthy older adults. Exp Gerontol.

[44] Smith-Ray RL, Hughes SL, Prohaska TR, Little DM, Jurivich DA, Hedeker D (2015). Impact of cognitive training on balance and gait in older adults. J Gerontol B Psychol Sci Soc Sci.

[45] Verghese J, Mahoney J, Ambrose AF, Wang C, Holtzer R (2010). Effect of cognitive remediation on gait in sedentary seniors. J Gerontol A Biol Sci Med Sci.

[46] Bruce H, Lai L, Bherer L, Lussier M, Onge NS, Li KZ (2019). The effect of simultaneously and sequentially delivered cognitive and aerobic training on mobility among older adults with hearing loss. Gait Posture.

[47] Wollesen B, Pocovi NC, Salvestro K, Hurley S, Seydell L, Scrivener K, Dean CM (2021). Multitask training to improve walking performance in older adults with hearing impairment: a feasibility study. J Aging Health.

[48] Nolan LS (2020). Age-related hearing loss: Why we need to think about sex as a biological variable. J Neurosci Res.

[49] Reavis KM, Bisgaard N, Canlon B, Dubno JR, Frisina RD, Hertzano R, Humes LE, Mick P, Phillips NA, Pichora-Fuller MK, Shuster B (2023). Sex-linked biology and gender-related research is essential to advancing hearing health. Ear Hear.

[50] World Health Organization. Report of the informal working group on prevention of deafness and hearing impairment Programme planning. Geneva: World Health Organization; 1991. p. 18-21. https://iris.who.int/handle/10665/58839?&locale-attribute=de.

[51] World Health Organization (2021). World report on hearing.

[52] Nasreddine ZS, Phillips NA, Bedirian V, Charbonneau S, Whitehead V, Collin I (2005). The Montreal Cognitive Assessment, MoCA: A brief screening tool for mild cognitive impairment. J Am Geriatr Soc.

[53] Carson N, Leach L, Murphy KJ (2018). A re-examination of Montreal Cognitive Assessment (MoCA) cutoff scores. Int J Geriatr Psychiatry.

[54] Ferris FL, Kassoff A, Bresnick GH, Bailey I (1982). New visual acuity charts for clinical research. Am J Ophthalmol.

[55] Ehrlich JR, Goldstein J, Swenor BK, Whitson H, Langa KM, Veliz P (2022). Addition of vision impairment to a life-course model of potentially modifiable dementia risk factors in the US. JAMA Neurol.

[56] Franchignoni F, Horak F, Godi M, Nardone A, Giordano A (2010). Using psychometric techniques to improve the Balance Evaluation Systems Test: The Mini-BESTest. J Rehabil Med.

[57] Anson E, Thompson E, Ma L, Jeka J (2019). Reliability and fall risk detection for the BESTest and Mini-BESTest in older adults. J Geriatr Phys Ther.

[58] Pothier K, Gagnon C, Fraser SA, Lussier M, Desjardins-Crépeau L, Berryman N, Kergoat MJ, Vu TM, Li KZH, Bosquet L, Bherer L (2017). A comparison of the impact of physical exercise, cognitive training and combined intervention on spontaneous walking speed in older adults. Aging Clin Exp Res.

[59] Bolia RS, Nelson WT, Ericson MA, Simpson BD (2000). A speech corpus for multitalker communications research. J Acoust Soc Am.

[60] Lau ST, Maracle J, Coletta D, Singh G, Campos J, Pichora-Fuller MK (2012). Auditory spatial attention in a complex acoustic environment while walking: Investigation of dual-task performance. Can Acoust.

[61] Campos JL, McCumber D, Chapnik B, Singh G, Lau ST, Li KZ, Nieborowska V, Pichora-Fuller K (2018). Perspectives on how acoustical, non-acoustical, and user characteristics should be considered in multimodal virtual reality research and application. Can Acoust.

[62] Wechsler D. Wechsler Adult Intelligence Scale WAIS - IV Canadian. San Antonio, TX: Pearson; 2008.

[63] Reitan RM, Wolfson D (1985). The Halstead-Reitan Neuropsychological Test Battery: Therapy and clinical interpretation.

[64] Delis DC, Kaplan E, Kramer JH (2001). Delis-Kaplan Executive Function System.

[65] Lezak MD, Howieson DB, Loring DW, Fischer JS (2004). Neuropsychological assessment.

[66] Tombaugh TN (2004). Trail Making Test A and B: Normative data stratified by age and education. Arch Clin Neuropsychol.

[67] Schmidt M. Rey Auditory Verbal Learning Test: A handbook. Los Angeles, CA: Western Psychological Services; 1996.

[68] Pelli DG, Robson JG, Wilkins AJ (1988). The design of a new letter chart for measuring contrast sensitivity. Clin Vis Sci.

[69] Giguère C, Lagacé J, Ellaham NN, Pichora-Fuller MK, Goy H, Bégin C (2020). Development of the Canadian Digit Triplet Test in English and French. J Acoust Soc Am.

[70] Bastianelli M, Mark AE, McAfee A, Schramm D, Lefrançois R, Bromwich M (2019). Adult validation of a self-administered tablet audiometer. J Otolarynol-Head-N.

[71] Zelinski EM, Gilewski MJ (2004). A 10-item Rasch modeled memory self-efficacy scale. Aging Ment Health.

[72] Smith SL, Kathleen Pichora-Fuller M, Watts KL, La More C (2011). Development of the listening self-efficacy questionnaire (LSEQ). Int J Audiol.

[73] Weinstein BE, Ventry IM (1983). Audiometric correlates of the Hearing Handicap Inventory for the elderly. J Speech Hear Disord.

[74] Powell LE, Myers AM (1995). The Activities-specific Balance Confidence (ABC) Scale. J Gerontol A Biol Sci.

[75] Arbuckle TY, Gold DP, Chaikelson JS, Lapidus S (1994). Measurement of activity in the elderly: The Activities Checklist. Can J Aging.

[76] Bassuk SS, Glass TA, Berkman LF (1999). Social disengagement and incident cognitive decline in community-dwelling elderly persons. Ann Intern Med.

[77] Jobsis F (1977). Noninvasive, infrared monitoring of cerebral and myocardial oxygen sufficiency and circulatory parameters. J Sci.

[78] Tadel F, Baillet S, Mosher JC, Pantazis D. Leahy RM. Brainstorm: A user-friendly application for MEG/EEG analysis. Comput Intell Neurosci. 2011;2011:879716. 10.1155/2011/879716 PMC3090754 21584256

[79] Mirelman A, Maidan I, Bernad-Elazari H, Shustack S, Giladi N, Hausdorff JM (2017). Effects of aging on prefrontal brain activation during challenging walking conditions. Brain Cogn.

[80] Ohsugi H, Ohgi S, Shigemori K, Schneider EB (2013). Differences in dual-task performance and prefrontal cortex activation between younger and older adults. BMC Neurosci.

[81] Fraser SA, Dupuy O, Pouliot P, Lesage F, Bherer L (2016). Comparable cerebral oxygenation patterns in younger and older adults during dual-task walking with increasing load. Front Aging Neurosci.

[82] Holtzer R, Mahoney JR, Izzetoglu M, Izzetoglu K, Onaral B, Verghese J (2011). fNIRS study of walking and walking while talking in young and old individuals. J Gerontol A Biol Sci Med Sci.

[83] Reuter-Lorenz PA, Park DC (2014). How does it STAC up? revisiting the scaffolding theory of aging and cognition. Neuropsychol Rev.

[84] Chambers LW, Bancej C, McDowell I (2016). Prevalence and monetary costs of dementia in Canada: population health expert panel.

[85] SMARTRISK. The economic burden of injury in Canada. Toronto, ON: SMARTRISK; 2009. Available from: http://www.parachutecanada.org/research/item/economic-burden-of-injury-reports.

